# Assembly and characterization of heterochromatin and euchromatin on human artificial chromosomes

**DOI:** 10.1186/gb-2004-5-11-r89

**Published:** 2004-10-27

**Authors:** Brenda R Grimes, Jennifer Babcock, M Katharine Rudd, Brian Chadwick, Huntington F Willard

**Affiliations:** 1 Department of Genetics, Center for Human Genetics, Case Western Reserve University School of Medicine and University Hospitals of Cleveland, Cleveland, OH 44106, USA; 2 Institute for Genome Sciences and Policy and Department of Molecular Genetics and Microbiology, Duke University, 103 Research Drive, Durham, NC 27710, USA; 3 Current address: Indiana University, School of Medicine, Department of Medical and Molecular Genetics, Medical Research Building 130, 975 West Walnut Street, Indianapolis, IN 46202-5251, USA

## Abstract

**Background:**

Human centromere regions are characterized by the presence of alpha-satellite DNA, replication late in S phase and a heterochromatic appearance. Recent models propose that the centromere is organized into conserved chromatin domains in which chromatin containing CenH3 (centromere-specific H3 variant) at the functional centromere (kinetochore) forms within regions of heterochromatin. To address these models, we assayed formation of heterochromatin and euchromatin on *de novo *human artificial chromosomes containing alpha-satellite DNA. We also examined the relationship between chromatin composition and replication timing of artificial chromosomes.

**Results:**

Heterochromatin factors (histone H3 lysine 9 methylation and HP1α) were enriched on artificial chromosomes estimated to be larger than 3 Mb in size but depleted on those smaller than 3 Mb. All artificial chromosomes assembled markers of euchromatin (histone H3 lysine 4 methylation), which may partly reflect marker-gene expression. Replication timing studies revealed that the replication timing of artificial chromosomes was heterogeneous. Heterochromatin-depleted artificial chromosomes replicated in early S phase whereas heterochromatin-enriched artificial chromosomes replicated in mid to late S phase.

**Conclusions:**

Centromere regions on human artificial chromosomes and host chromosomes have similar amounts of CenH3 but exhibit highly varying degrees of heterochromatin, suggesting that only a small amount of heterochromatin may be required for centromere function. The formation of euchromatin on all artificial chromosomes demonstrates that they can provide a chromosome context suitable for gene expression. The earlier replication of the heterochromatin-depleted artificial chromosomes suggests that replication late in S phase is not a requirement for centromere function.

## Abstract 2

An assay of the formation of heterochromatin and euchromatin on *de novo* human artificial chromosomes containing alpha satellite DNA revealed that only a small amount of heterochromatin may be required for centromere function and that replication late in S phase is not a requirement for centromere function.

## Background

In the post-sequencing phase of genome characterization, it is important to understand the contribution of non-coding sequences to higher-order genome structure and stability. Maintenance of genome integrity and the faithful transmission of genetic information in mitosis and meiosis are essential to organism survival and are critically dependent on two repetitive chromosomal elements. Telomeres protect against chromosomal truncation or fusion events [1], while centromeres ensure faithful chromosome segregation through cell division [2-4]. Failure in the function of these elements can lead to genomic instability, with often catastrophic consequences in humans such as miscarriage, congenital birth defects or cancer. In contrast to the telomere, whose properties have been well explored at the genomic and molecular levels [5], the human centromere remains relatively poorly characterized, and experimental systems for the genomic study of centromere formation and behavior are only just being developed and optimized [6-14].

Defining the minimal DNA sequences required for centromere function on a normal human chromosome has proved challenging, owing to the complex nature of inter- and intra-chromosomal homology and variability in genomic DNA content near the primary constriction. Common to all normal human centromeres are large amounts of alpha-satellite DNA, which is comprised of a family of diverged 'monomers' of around 171 base-pairs (bp) that have been amplified in multimeric groups (higher-order repeats) on different chromosomes to form chromosome-specific arrays typically megabases in length [15-17]. In addition, the core of higher-order repeat alpha-satellite is, where examined in detail, surrounded by other alpha-satellite sequences that fail to form a recognizable higher-order structure (so-called 'monomeric' alpha satellite) [10,18-20]. Together, the two types of centromeric repeat span up to several megabases of genomic DNA at each centromere region and account for much of the largest remaining gaps in the human genome sequence assembly [21,22]. Support for a critical role for alpha-satellite DNA in centromere function comes from recent studies on the human X chromosome, where the most abundant alpha-satellite sequence at this centromere, DXZ1, has been shown to be sufficient for centromere function [10,23] and, more generally, from studies demonstrating the formation of *de novo *centromeres on human artificial chromosomes following transfection of some types of alpha-satellite sequences into human cells [6-14].

Paradoxically, despite conservation of the functional role of the centromere in every eukaryotic cell, DNA sequences at eukaryotic centromeres are quite divergent in sequence even between closely related species [24,25]. Although primary genomic sequence has not been conserved at eukaryotic centromeres, they do, nonetheless, share features in common such as a structure based on tandem repeats, overall AT-rich composition, and packaging into specialized centromeric chromatin marked by the presence of centromere-specific histone H3 (CenH3) variants (reviewed in [4,26,27]). The ability of different genomic sequences to fulfill centromeric requirements in different species is in accord with data showing that the DNA normally associated with the genetically mapped centromere on normal human chromosomes is not always sufficient or necessary for centromere function. Rare chromosomal rearrangements can result in either dicentric chromosome formation, where one centromere is typically inactivated [28,29], or in the formation of neocentromeres, where a centromere assembles on DNA that is not associated with the normal centromere genomic locus (reviewed in [3]). Together, these observations suggest that epigenetic factors are critical for centromere function [30] and point to the as-yet incompletely understood interplay of underlying genomic DNA sequences located in the centromeric region and their ability to package into specialized centromeric chromatin [2,4,27].

Recent evidence suggests that a complex system of epigenetic modifications based on histone variants and histone tail modifications is important for centromere activity (reviewed in [4,31]), in much the same way as a histone code is involved in determining the transcriptional competence of DNA [32]. Although the epigenetic basis of centromere function is not yet fully defined, a strong candidate for specifying the site of the functional centromere (kinetochore-forming region) is the family of CenH3 variants, which are conserved from yeast to humans and are essential to viability of the organism (reviewed in [2]). In humans and flies, CenH3 is restricted to the centromere where CenH3- and typical H3-containing nucleosomes exist in an alternating arrangement, generating a unique chromatin structure that may be important for centromere function [33,34].

The most completely studied complex eukaryotic centromere at the molecular level is that of the fission yeast *Schizosaccharomyces pombe*. Detailed analyses of a 40-kilobase (kb) *S. pombe *centromere revealed that it encompasses both the kinetochore, as defined by the exclusive association of Cnp1, the fission yeast CenH3, with the central core element [35] and adjacent repeats enriched for heterochromatin-associated factors [36] that are important for centromeric cohesion [37-40]. Within the heterochromatic domains, histone H3 is methylated at lysine 9 (H3MeK9), resulting in the recruitment of the heterochromatin protein HP1-homolog Swi6 [41].

There is substantial evidence that HP1 is involved in setting up and/or maintaining a repressed chromatin state in several epigenetic systems (reviewed in [42]). HP1 proteins are conserved and localize to centromere regions in human and mouse cells [43-45]. Human cells express three HP1 isoforms, HP1α, HP1β and HP1γ. HP1α and HP1β localize primarily to pericentromeric regions, while HP1γ is dispersed at sites along chromosome arms [43]. Furthermore, modified H3MeK9 nucleosomes, which create a binding site for HP1 (reviewed in [46]), have also been localized cytologically to centromere regions in flies and mice [44,47-53]. These observations suggest a model in which local modifications of chromatin composition represent a crucial and highly conserved element necessary for the specification and/or maintenance of complex eukaryotic centromeres [2]. Consistent with these models, chromatin immunoprecipitation assays with highly specific antibodies have shown that both mouse minor and major satellite DNA sequences exhibit trimethylation of histone H3 at lysine 9 [51,53]. However, while the association of histone modifications typical of repressive heterochromatin has been clearly demonstrated for sequences that flank the functional centromere, it is less certain what modifications, if any, may characterize the CenH3-containing chromatin of the functional centromere itself. Indeed, many of the characteristics historically assigned to pericentromeric DNA (that is, repressive heterochromatin and late-replication in S phase [54,55]) may be features of the surrounding heterochromatin, more so than of the functional centromere *per se*.

One way to address the interacting and complementary role(s) of DNA sequence and *trans*-acting chromatin factors in human centromere function is through the construction of detailed genomic maps of human centromeric regions and evaluation of their associated proteins [10,19,56,57]. An alternative empirical approach is to construct minimal human artificial chromosomes from defined alpha-satellite DNA sequences [6-14] as tools for evaluating the essential genomic requirements of centromere specification. Indeed, previous studies have shown that the human CenH3 - centromere protein A (CENP-A) - is deposited at the centromere on artificial chromosomes constructed from alpha-satellite DNA [12,13,58]. However, it is not known whether heterochromatin formation is required for centromere establishment and propagation and/or whether *de novo *centromeres on human artificial chromosomes without large amounts of adjacent heterochromatin demonstrate the same chromatin characteristics as either normal human centromeres or human artificial chromosomes with large amounts of heterochromatin.

In the present study, we have characterized the nature of heterochromatin and euchromatin formed on a series of human artificial chromosomes derived from higher-order repeat alpha satellite from chromosomes X or 17 [12,14]. While large artificial chromosomes contain substantial amounts of heterochromatin (characterized by the presence of modified H3MeK9 nucleosomes and HP1α) and replicate later in S phase, small artificial chromosomes show features more consistent with the euchromatin of the chromosome arms, including the presence of histone variants typical of expressed euchromatin and replication earlier in S phase. These data suggest that the chromatin environment required for *de novo *centromere formation and function is likely to be generally conducive to gene expression, as will probably be required for either gene-transfer experiments and/or functional genomic applications of the artificial chromosome technology. Further, the data raise the possibility that functional centromeres may adopt a novel chromatin state that is, contrary to what has been long assumed, quite distinctive from that of conventional heterochromatin.

## Results

To examine the chromatin composition of human artificial chromosomes, we used a panel of artificial chromosomes formed after transfection with vectors containing either synthetic chromosome 17 (D17Z1) or cloned X chromosome (DXZ1) alpha-satellite sequences [12,14]. Each of the artificial chromosomes tested contains a functional *de novo *centromere assembled from the transfected DNA, as well as at least one copy of a functioning gene used as a selectable marker. Together, this panel of artificial chromosomes provides an opportunity to examine the nature of heterochromatin and euchromatin assembled on the transfected DNA sequences. The high mitotic stability and *de novo *composition of artificial chromosomes generated from D17Z1 (17-E29, 17-D34 and 17-B12) or DXZ1 (X-4 and X-5) have been described [12,14]. As a more direct measure of artificial chromosome segregation errors, we have used an assay that allows cells to undergo anaphase but cannot complete cytokinesis [14]. Using fluorescence *in situ *hybridization (FISH), artificial and host chromosome segregation products can be measured and nondisjunction or anaphase lag defects recorded.

In X-4 and X-5, artificial chromosomes mis-segregated in 1.8% and 2.4% of cells, respectively ([14] and Table 1). Similar analyses of artificial chromosome segregation errors in 17-B12 revealed that they mis-segregated in 2.4% of the cells (Table 1). This segregation error rate is comparable to that found for the majority of other human artificial chromosomes previously characterized [14]. Artificial chromosomes in 17-E29 and 17-D34 have segregation efficiencies corresponding to more than 99.9% per cell division, using metaphase analyses [12]. For comparison, we also examined an additional cell line, 17-C20, which contains highly mitotically unstable D17Z1-based artificial chromosomes. In 17-C20, artificial chromosome copy number was high (average 4.7 per cell) and artificial chromosomes were lost from the cell population by 30-40 days of culture without selection, despite containing both inner (CENP-A) and outer (CENP-E) kinetochore proteins (data not shown). In the anaphase assay, 12.2% of artificial chromosomes in 17-C20 were mis-segregating (at 12 days without selection) and the predominant defect was anaphase lag (Table 1). Sizes of D17Z1-containing artificial chromosomes were based on comparison of the signal intensity on the approximately 3 Mb D17Z1 array on chromosome 17 to intensities on the artificial chromosomes using FISH analyses with a D17Z1 probe (Table 2; see also Figures 2 and 3 in [12]). Artificial chromosomes that had signal intensities several-fold less than the endogenous D17Z1 signals were estimated to be 1-3 Mb in size, whereas artificial chromosomes that produced signals similar to or several-fold more intense than those of the endogenous D17Z1 arrays were estimated to be in the 3-10 Mb size range. Similar comparisons of the signal intensities on the DXZ1-based artificial chromosomes with those of the host DXZ1 signals were used to estimate the sizes of the DXZ1-based human artificial chromosomes (Table 2 and data not shown). Properties of artificial chromosomes used in the present study are summarized in Tables 1 and 2.

### Variation in levels of heterochromatin-associated factors correlates with artificial chromosome size

**Figure 1:**
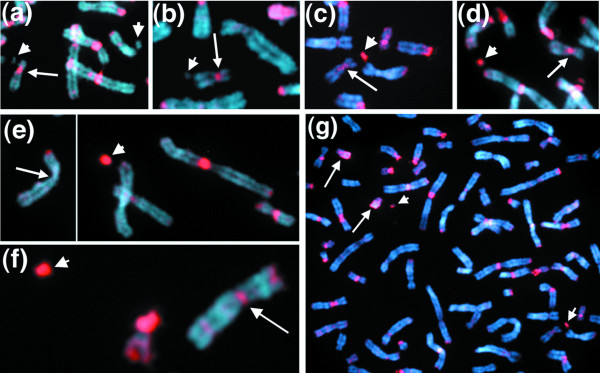
Heterochromatin forms on artificial chromosomes in the 3-20 Mb size range but is depleted on smaller artificial chromosomes that are approximately 1-3 Mb. Indirect immunofluorescence using an antibody that recognizes modification of histone H3 by trimethylation at lysine 9/lysine 27 (H3TrimK9/K27) (red signal) demonstrated that these heterochromatin markers are not detectable on the smaller D17Z1-based artificial chromosomes (arrowheads) in lines **(a) **17-D34 and **(b) **17-E29, but are readily detectable on the larger D17Z1- and DXZ1-based artificial chromosomes (arrowheads) as shown in lines **(c) **17-B12, **(d) **17-C20, **(e) **X-4 and **(f) **X-5. Arrows indicate chromosome 17 centromere regions (a-d) or host X centromere regions (e, f). Host D17Z1 sequences typically stained positive for H3TrimK9/K27 in most spreads (arrows in a-d). It was difficult to detect the X centromere signal (for example, arrow in (e)) but in about 30% of spreads there was a clearly positive signal as indicated by the arrow in (f). **(g) **Variation in H3TrimK9/K27 levels at host centromere regions is shown in a larger area of the spread shown in (c): artificial chromosomes are indicated by arrowheads; arrows point to the consistently strongly positive signals on the long arm of the Y chromosome (Yq). Artificial chromosome size estimates are listed in Table 2. Confirmation of artificial chromosomes and relevant host centromere regions were determined by FISH analyses with appropriate alpha-satellite probes (data not shown).

To test whether human artificial chromosomes were capable of forming heterochromatin, we first examined several established markers of heterochromatin on the artificial chromosome panel. Indirect immunofluorescence with an antibody recognizing histone H3 modified by trimethylation at lysine 9 and lysine 27 (H3TrimK9/K27) was applied to metaphase spreads. Methylation of lysines at these sites has been associated with formation of repressive chromatin, including pericentric heterochromatin in mouse cells [32,51-53,59,60]. As shown in Figure 1a and 1b, small D17Z1-based artificial chromosomes, estimated to be in the 1-3 Mb size range (Table 2), do not stain detectably with the H3TrimK9/K27 antibody, in contrast to the centromeric regions of the natural human chromosomes that stain, in some cases intensely, with this antibody. On the other hand, larger artificial chromosomes, estimated to be in the 3-20 Mb size range (Table 2), stained strongly for H3TrimK9/K27 modifications (Figure 1c-g), often at levels greater than those of many endogenous centromeric regions (Figure 1g). It is clear that at least large amounts of transfected alpha satellite are capable of assembling into heterochromatin in the context of human artificial chromosomes. Whether small artificial chromosomes are truly negative for this marker of heterochromatin, or whether they assemble only small amounts of heterochromatin below the level of detection, cannot be assessed with this assay. Nonetheless, they clearly have assembled far less of this epigenetically modified heterochromatin than exists at the relevant endogenous 17 centromeric regions (Figure 1).

**Figure 2:**
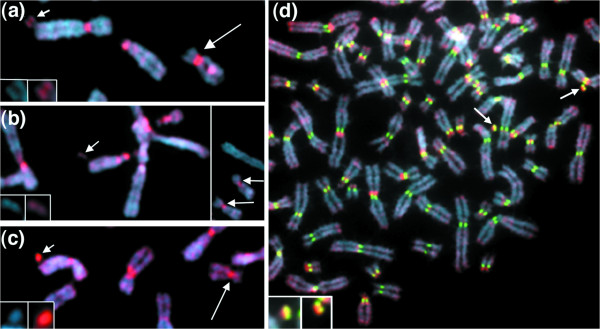
Detection of HP1α on D17Z1-based artificial chromosomes. **(a-d) **Cell lines stably expressing a Myc-tagged form of HP1α. HP1α was detected using an anti-Myc antibody (red). The artificial chromosomes (about 1-3 Mb; indicated by small arrows) in lines **(a) **17-D34-1.A2 and **(b) **17-E29-1.C23 exhibit faint HP1α staining at a level similar to the general arm staining. Larger artificial chromosomes (3-10 Mb; small arrow) in lines **(c) **17-C20-1.B22 and **(d) **17-B12-1.B10 stain strongly for HP1α. Inserts in (a-c) show either DAPI (blue)-stained artificial chromosomes or HP1α (red). Host 17 centromere regions are indicated by the large arrows in (a-c). In (d), simultaneous staining for CENP-A (green) shows that CENP-A is restricted to a portion of the artificial chromosome (arrows) whereas the HP1α signal coats the entire artificial chromosome. In contrast to CENP-A, which is present at comparable levels on all artificial chromosomes tested [12,13,58] and host kinetochores [61], HP1α staining levels are more variable at host centromere regions (d).

In a parallel approach, we examined the distribution of HP1α in four lines containing D17Z1-based artificial chromosomes. Each line was stably transfected with a Myc-epitope tagged form of HP1α (see Materials and methods) to permit detection of HP1α using an anti-Myc antibody. The smaller artificial chromosomes stained very weakly (at a level similar to that of the staining on the euchromatic chromosome arms), well below the levels of HP1α detected at the centromeric region of the endogenous chromosome 17s (Figure 2a,b). As seen with the H3TrimK9/K27 antibody, the larger artificial chromosomes stained strongly for HP1α (Figure 2c,d), at levels comparable to the endogenous chromosome 17s. The intensity of HP1α-Myc staining was variable at endogenous human centromere regions (Figure 2d); similar results were obtained using a primary anti-HP1α antibody (data not shown). This contrasts with the amount of CENP-A, which appears to be present at consistent levels at all normal human centromeres [61] and artificial chromosomes tested (Figure 2d) [12,13,58]. Notably, the CENP-A signal is localized to a discrete subdomain within the larger artificial chromosomes, whereas HP1α covers a much larger area of the artificial chromosome (Figure 2d). This suggests that HP1α may be a marker for generalized pericentromeric heterochromatin that flanks the kinetochore-associated alpha satellite of the functional centromere, rather than a marker of the functional centromere *per se*. Such a model [2,3] is also consistent with the observation that small artificial chromosomes, which contain little if any of the flanking heterochromatin, do not contain elevated levels of HP1α (Figure 2a,b; Table 2).

### Euchromatin forms on artificial chromosomes

For their potential use as gene-transfer vectors or as general vehicles suitable for interrogation of genome function, human artificial chromosomes must also be capable of forming euchromatin to support gene expression. Indeed, one would hypothesize that at least small amounts of transcriptionally active chromatin must form during artificial chromosome formation to permit expression of the selectable marker gene(s) contained on the transfected constructs [10,12,14]. It has previously been shown using immunocytochemical methods [62,63] that methylation of histone H3 at lysine 4, an epigenetic modification associated with transcriptionally permissive chromatin [64-66], is generally enriched on autosomes and depleted at the repressed inactive X chromosome and human centromere regions.

**Figure 3:**
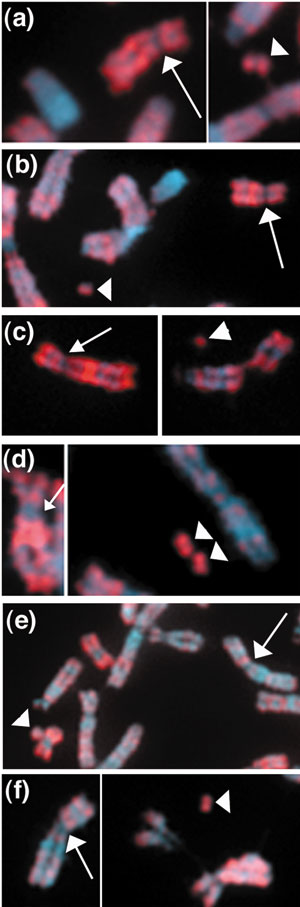
Transcriptionally competent chromatin is present on artificial chromosomes. Dimethylation of lysine 4 on histone H3 (H3DimK4) was visualized using an antibody against H3DimK4 (red). This euchromatin mark was detected on all artificial chromosomes (arrowheads) generated from either D17Z1 in lines **(a) **17-D34, **(b) **17-E29, **(c) **17-B12 and **(d) **17-C20, or DXZ1 in lines **(e) **X-4 or **(f) **X-5. Host centromere regions were generally depleted for H3DimK4 as indicated by arrows pointing to centromere regions of chromosome 17 (a-d) and the X chromosome (e, f).

As a test for formation of permissive chromatin on artificial chromosomes, we stained metaphase spreads with an antibody that recognizes histone H3 dimethylated at lysine 4 (H3DimK4). All artificial chromosomes tested stained positively for H3DimK4 modifications (Figure 3a-f; Table 2). In contrast, the endogenous centromeric regions were depleted for H3DimK4 staining, although, as noted above for markers of heterochromatin formation, this depletion may reflect the state of the surrounding heterochromatin, rather than that of the functional centromere *per se*.

Previous structural analyses of artificial chromosomes indicate that they consist of input DNA multimers arranged as blocks of alpha-satellite DNA interspersed with vector sequences [7,11,12]. This structural organization is consistent with the presence of multiple selectable marker genes and differs from the large uninterrupted blocks of alpha-satellite DNA found at all human centromeres that are typically under-represented for this active chromatin mark (Figure 3). Because mitotically stable artificial chromosomes can have permissive as well as repressive chromatin present, these data suggest that this chromatin configuration does not significantly disturb mitotic centromere function.

### Two modes of artificial chromosome replication timing

While the genomic determinants of potential origins of DNA replication in the human genome, as well as of their timing of replication during S phase, are still not well understood, the generally accepted paradigm is that expressed sequences replicate in the first half of S phase, while non-expressed sequences replicate in the second half [67]. Consistent with this pattern, alpha-satellite DNA, as well as constitutive heterochromatin (such as that found on the Yq arm), replicate in the mid to late S phase period [54,55,68,69]. In the present study, we have asked whether D17Z1-based artificial chromosomes replicate at a similar time to endogenous chromosome 17 alpha-satellite DNA. To determine the time of replication, unsynchronized cells were pulsed with bromodeoxyuridine (BrdU) for 2 hours, followed by a thymidine chase for varying lengths of time before harvesting cells in metaphase (see Materials and methods). Detection of BrdU incorporation at sites of DNA replication was performed using indirect immunofluorescence with an anti-BrdU antibody on metaphase spreads.

**Figure 4:**
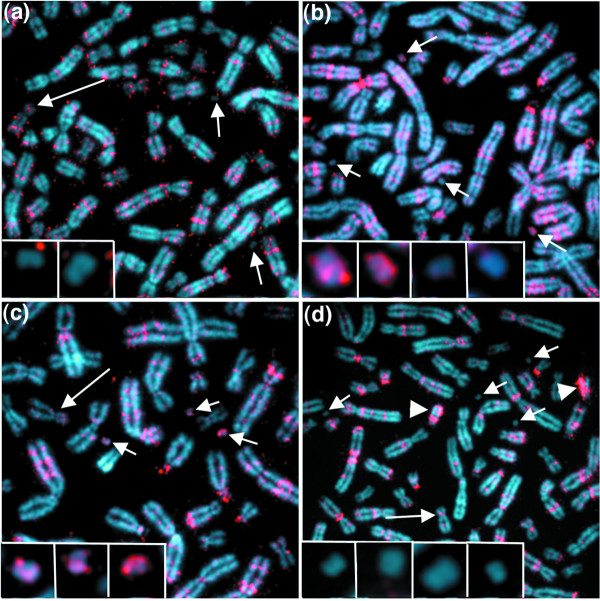
Replication timing of human artificial chromosomes in line 17-B12. BrdU detection (red) in cells that have been blocked with colcemid in mitosis following BrdU pulses during S phase (see Materials and methods). Artificial chromosome (small arrows; enlarged artificial chromosomes are shown in inserts) and chromosome 17 (large arrow) locations in each spread were confirmed by FISH analyses using a D17Z1 probe (data not shown). **(a-d) **Images from different periods in S phase. (a) Early in S phase, at 0-2 h, the two artificial chromosomes present in this spread are not replicating. Some incorporation of BrdU on chromosome 17 is detectable. (b) In the middle of S phase, at 2-4 h, two of four artificial chromosomes are replicating. (c) Later, at 4-6 h, all three artificial chromosomes are being coordinately replicated. Some BrdU incorporation within chromosome 17 arms is detectable. (d) Late in S phase, at 6-8 h, artificial chromosomes are not replicating. The centromere region on chromosome 17 is replicating (large arrow). Because of the A-rich sequence composition of satellite III on Yq, BrdU is preferentially incorporated into one strand, producing an asymmetrical staining pattern on Yq (arrowheads) [84].

**Figure 5:**
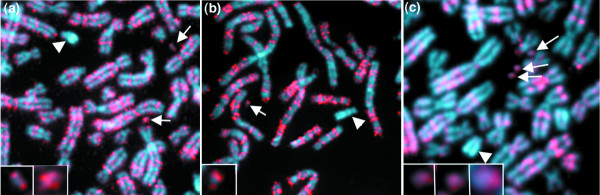
Replication timing in different human artificial chromosomes. **(a-c) **Detection of BrdU (red) on artificial chromosomes (small arrows; larger version in inserts). (a) In mid S phase, at 2-4 h, two artificial chromosomes in line 17-D34 are BrdU positive. (b) The artificial chromosome in line 17-E29 is replicating early in S phase, in the 0-2 h period. (c) In mid S phase (2-4 h), three artificial chromosomes are being coordinately replicated in this spread from line 17-C20. Images shown are from the first half of S phase, and, as expected, Yq (arrowhead) is not replicating at this time.

While there was overlap between artificial chromosome replication timing patterns and those of the host 17 centromere regions during mid S phase (Table 3), we found two modes of artificial chromosome replication timing. The heterochromatin-enriched artificial chromosomes (17-B12 and 17-C20; see Table 2) commenced replication in mid S phase (2-4 hours into S phase) and completed replication by 6 hours into S phase (Figures 4 and 5c; Table 3). In contrast, the heterochromatin-depleted artificial chromosomes (17-D34 and 17-E29; see Table 2) started replicating within the first 2 hours of S phase (early S phase) and their replication was completed by 4 hours into S phase (Figure 5a,b; Table 3). That these differences are characteristic of each particular artificial chromosome is suggested by the observation that, in all lines, when multiple artificial chromosomes were present in a given cell, they are frequently replicated synchronously (Figures 4c and 5a,c). From these data, it is tempting to propose that the presence of large amounts of heterochomatin in the larger artificial chromosomes may have influenced replication timing on these artificial chromosomes and promoted a shift towards later in S phase.

## Discussion

Human artificial chromosomes provide a novel system for analyzing *cis*- and *trans*-acting factors necessary for chromosome segregation and offer potential for both functional genomics and gene-transfer applications. The artificial chromosomes we used contain defined alpha-satellite DNA sequences [12,14]. Studying how epigenetic components assemble with alpha satellite to form a *de novo *centromere on artificial chromosomes may reveal the critically important components and may help distinguish between those features that are characteristic of the functional centromere itself and those that are markers of the surrounding heterochromatin. Such a distinction is extremely difficult in normal human chromosomes but should be enhanced by the ability to generate a variety of different artificial chromosomes made with different input sequences.

Recent detailed molecular studies in the fission yeast have revealed that such epigenetic factors are critical for centromere function. The fission yeast CenH3, Cnp1, is deposited only at the central core domain, while heterochromatin (marked by methylation of histone H3 at lysine 9 and by binding of the HP1 homolog, Swi6) forms on the surrounding inverted repeats [35,36,41]. The yeast data, together with the observations that CenH3s are conserved and that H3K9-modified nucleosomes and HP1 proteins are often found close to the centromere in higher eukaryotes, have contributed to the development of models for centromere packaging in the larger chromosomes of multicellular eukaryotes, including mammals. In these models, a specific centromeric chromatin configuration, in which CenH3-containing chromatin is surrounded by pericentric heterochromatin, is conserved and may be an important determinant of centromere function [2-4].

While the data presented here are largely consistent with these models, they permit two important refinements. First, large amounts of heterochromatin (containing alpha satellite and marked by H3TrimK9/K27 staining, HP1α binding and late replication) are not required for effective chromosome segregation during mitosis; indeed, the small artificial chromosomes examined here do not contain detectable amounts of H3TrimK9/K27 (Table 2). Second, the cytological characteristics of heterochromatin (repressive chromatin and later replication in S phase), classically attributed to the centromere [54,55], may instead reflect features of the surrounding heterochromatin and do not appear to define critical properties of the functional centromere. Our own data would argue that the functional centromere - at least as assembled on the smaller D17Z1-based human artificial chromosomes - is instead characterized by a distinctive chromatin containing CenH3 (CENP-A) that can form within regions epigenetically modified with markers of euchromatin (Tables 1 and 2). This conclusion is consistent with parallel work on the organization of centromeric chromatin of normal *Drosophila *and human chromosomes [34]. The finding that CENP-A-containing chromatin can be deposited within euchromatin-rich artificial chromosomes that are highly mitotically stable (more than 99.9 % segregation efficiency per cell division) yet depleted for heterochromatin modifications, suggests that only a very small amount of heterochromatin may be required on an artificial chromosome (from observations in yeast [37-40] and chicken DT40 cells [70] this is presumably for assembling the cohesin complex), and that this could also be true for human centromeres.

This study also addresses the question of timing of replication of D17Z1-based artificial chromosomes. The smaller artificial chromosomes that completely overlap with CENP-A [12] and euchromatic modifications (Figure 3) replicate early in S phase whereas the larger artificial chromosomes that have assembled heterochromatin (H3TrimK9/K27 and HP1α) in addition to euchromatin replicate later in S phase (Table 3). The later onset of replication on the larger artificial chromosomes is similar to that of host chromosome 17 centromere regions that are also enriched for H3TrimK9/K27 and HP1α (Figures 1 and 2, Tables 2 and 3). With the caveats that higher-resolution methods will be required to determine the precise replication timing of the CENP-A domain on the artificial chromosomes, and that differences in vector DNA content may be influencing origin establishment and/or usage, our observations are consistent with local chromatin modification being an important factor influencing artificial chromosome replication.

Chromatin composition as a factor in determining replication timing has also been implicated in a study of a *Drosophila *minichromosome deletion series. In this study, replication timing was shifted to an earlier point in mid-S phase following deletion of large amounts of pericentromeric heterochromatin from the minichromosomes [71]. Support for a direct role of chromatin composition in replication timing comes from studies in budding yeast, where regions associated with acetylated histones (an epigenetic mark of active chromatin) replicate earlier than those depleted for this histone modification [72]. However, unexpected recent evidence from fission yeast has shown that centromeric heterochromatin replicates early in S phase, suggesting that chromatin composition is not a uniform determinant of replication timing in lower eukaryotes [73]. As the euchromatin-rich and highly mitotically stable artificial chromosomes replicate in the first half of S phase (in 17-E29, the majority of artificial chromosomes (75%, *n *= 20) replicated in the first 2 hours of S phase (Table 3)) these findings challenge the current dogma that replication later in S phase is an obligatory function of the centromere. The present findings are also supportive of earlier studies suggesting that replication timing of CenH3-containing chromatin is not a determinant of the functional centromere [69,71].

Cytological data indicate that the amount of CENP-A modified chromatin (in addition to several other kinetochore-associated CENPs) is similar on endogenous human chromosomes and on all artificial chromosomes regardless of the amount of total alpha satellite present. This suggests that the amount of CENP-A chromatin and/or the size of the kinetochore is regulated and/or limited in some manner [6-14,58,61]. In contrast, the results of the present study indicate that the heterochromatic fraction of centromeric DNA (on both endogenous chromosomes and artificial chromosomes) is highly variable. In line with current models, we did detect elevated levels of H3TrimK9/K27 modifications and HP1α, diagnostic of heterochromatin on large artificial chromosomes generated from chromosome 17 (D17Z1) or X (DXZ1) alpha-satellite DNA. However, no immunocytochemically detectable heterochromatin (H3TrimK9/K27) was associated with the smaller artificial chromosomes.

To evaluate their potential for characterization of genome sequences and, eventually, for gene transfer or gene therapy applications, we sought to determine the extent of transcriptionally competent chromatin formation in artificial chromosomes. Epigenetic modification of histone H3 by dimethylation at lysine 4 (H3DimK4), a marker of transcriptionally competent chromatin, was present on all artificial chromosomes tested. This contrasts with the staining pattern associated with the centromere regions on human metaphase spreads, where this modification is largely undetectable, probably reflecting the general absence of genes mapping to centromere regions (Figure 3). As selectable marker genes are expressed on artificial chromosomes, it may be presumed that at least a portion of the artificial chromosome chromatin structure is transcriptionally permissive, consistent with the positive staining for H3DimK4. In line with these observations, large human transgenes have been expressed from artificial chromosomes [74-76] and selectable marker genes on artificial chromosomes assemble acetylated histones, another marker of euchromatin [77]. Furthermore, detection of transcription of genes within the CenH3 domain of a human neocentromere [78] and a rice centromere [79] suggests that CenH3-containing chromatin can be transcriptionally competent. The relationship between active and repressive chromatin and underlying genomic sequences on the larger artificial chromosomes is not known and will require more detailed follow-up analyses. As other detailed chromatin immunoprecipitation studies have shown that methylation of histone H3 at lysine 4 or lysine 9 seem to be mutually exclusive [64,65], it will be interesting to find out how the two types of chromatin are assembled during artificial chromosome formation and to find out if there is a mechanism that prevents spreading of chromatin between the heterochromatic and euchromatic sub-domains. An advantage of the artificial chromosome system is the capacity to manipulate sequence content and to test directly the involvement of candidate sequences in gene expression, chromatin establishment or timing of DNA replication.

In this study we included one line, 17-C20, that contains *de novo *D17Z1-based artificial chromosomes that retain both inner and outer kinetochore components yet are highly mitotically unstable as a result of their rapid loss in the absence of selection and the very high segregation error rate (12.2%) detected in the anaphase assay (Table 1). The artificial chromosomes in this line have a global chromatin composition indistinguishable to that of similar-sized D17Z1-based mitotically stable artificial chromosomes, as both H3DimK4- and H3TrimK9/K27-modified nucleosomes and HP1α are assembled (Table 2). Our study has not revealed the cause of the segregation defect of artificial chromosomes in 17-C20, and so a more extensive examination of additional epigenetic markers or centromere-associated factors may be informative. Detailed anaphase segregation analyses of D17Z1- and DXZ1-based artificial chromosomes have revealed that there is a range of mitotic stability among artificial chromosomes [14]; future studies will aim to characterize the mechanistic basis of the segregation defects and the relative contribution of genomic and/or epigenetic factors to chromosome behavior.

## Conclusions

In summary, we have shown that artificial chromosomes assemble transcriptionally permissive chromatin and that there is a link between artificial chromosome size and the assembly of heterochromatin. Our results with the artificial chromosome panel are largely consistent with current models proposing that the formation of heterochromatin within the vicinity of CENP-A chromatin is functionally important, although the amount of heterochromatin assembled is quite variable, suggesting either that it is required only in small amounts or that it perhaps could even be dispensable. Strikingly, the studies here on the chromatin composition of artificial chromosomes, in combination with studies on normal human centromeres [34], strongly suggest that the chromatin state of the functional centromere region (as defined by CenH3 association) is quite distinct from pericentric heterochromatin. The artificial chromosome system provides a new set of reagents for investigating the role of both defined alpha-satellite DNA sequences and *trans*-acting epigenetic factors that cooperate to form a functional human centromere. A fuller understanding of the structure-function relationships of the chromatin and DNA composition of artificial chromosomes is important not only to further our understanding of the role of centromeres in genome stability, but also for the potential development of artificial chromosomes for gene transfer applications.

## Materials and methods

### Cell lines

Characterization of cell lines containing mitotically stable human artificial chromosomes formed after transfection with either synthetic D17Z1 arrays (PAC17HT1.E29 (17-E29), PAC17HT1.D34 (17-D34), BAC17HT4.B12 (17-B12) or cloned DXZ1 sequences (X-4, X-5) have been described previously [12,14]. The artificial chromosomes in 17-C20 were generated using VJ104-17α32 [12], hybridize with both D17Z1 and BAC vector probes, are *de novo *in composition and assemble CENP-A and CENP-E (data not shown). All artificial chromosomes were formed in human HT1080 cells. Cell lines were grown as described [12] and supplemented with either 100 μg/ml G418 (Gibco) (17-B12, 17-C20) or 2 μg/ml Blasticidin S HCl (ICN) (17-E29, 17-D34, X-5, X-6), as described [12].

### Anaphase assays

Anaphase assays used to directly measure chromosome segregation defects in 17-B12 and 17-C20 (Table 1) were carried out as previously described [14]. Assays were carried out at either 45 days (17-B12) or 12 days (17-C20) culture without selection. The spectrum orange-labeled D17Z1 probe (Vysis) hybridized with host 17 centromere regions and artificial chromosomes, whereas the spectrum green-labeled BAC vector probe VJ104 [6] hybridized exclusively with the artificial chromosomes. Co-localization of vector and D17Z1 probes produced yellow fluorescence on the artificial chromosomes, which allowed them to be distinguished from the host D17Z1 sequences (data not shown).

### Generation of clonal lines expressing Myc-tagged HP1α

The nucleotide sequence of human HP1α (NCBI Nucleotide database: S62077) was used in BLAST searches against entries in the human expressed sequence tag (EST) database using the NIH BLAST server [80]. A representative HP1α cDNA clone (IMAGE 627533) was obtained from Research Genetics. DNA was prepared with the Wizard-plus mini-prep DNA purification system (Promega), and the cDNA was sequenced on an ABI 373 (Perkin-Elmer) with a fluorescence labeled dye-terminator cycle sequencing kit according to the manufacturer's instructions (PRISM Ready DyeDeoxy Terminator Premix from Applied Biosystems). The full coding sequence of IMAGE 627533 was PCR-amplified with primers incorporating an *Eco*RI restriction enzyme recognition site (HP1α forward primer, 5'-GGAATT CTGATGGGAAAGAAAACCAAGCG-3'; reverse primer, 5'-GGAATTCGCTCTTTGCTGTTT CTTTC-3') and subcloned using standard techniques [81] into pcDNA3.1-CT-Myc-His (Invitrogen). Subclones were sequenced to verify sequence integrity and orientation as above. The HP1α-Myc tagged construct (pHP1α-Myc) was transfected into 17-C20, 17-B12, 17-E29 or 17-D34 cell lines using lipofectamine (Invitrogen), resulting in the formation of clonal lines (17-C20-1.B22, 17-B12-1.B10, 17-E29-1.C23 and 17-D34-1.A2, respectively) that stably express Myc-tagged HP1α. G418 selection at 400 μg/ml was applied to select clonal lines 17-E29-1.C23 and 17-D34-1.A2. Since 17-C20 and 17-B12 cells are G418-resistant, pHP1α-Myc was co-transfected in the presence of a second construct, pPAC4 [82] that carries a *bs*^r ^marker gene. Clonal lines (17-C20-1.B22 and 17-B12-1.B10) resistant to 4 μg/ml Blasticidin S HCl (ICN) were selected and expanded. Confirmation of Myc-tagged HP1α expression was by immunofluorescence using a mouse monoclonal anti-Myc antibody (Invitrogen).

### Immunofluorescence and fluorescence *in situ *hybridization (FISH)

Metaphase spreads were prepared for immunofluorescence using previously described protocols [29]. Primary antibodies to the dimethylated form of histone H3 at lysine 4 (anti-H3DimK4) were purchased from Upstate Biotechnology (anti-dimethyl-histone H3 (Lys4)). Modification of histone H3 by trimethylation at lysine 9 (H3TrimK9) was detected using an antibody to the tri-methylated form of histone H3 at lysine 9 purchased from Abcam (anti histone H3-tri methyl K9). This antibody cross-reacts with lysine 27 on histone H3 and is termed anti-H3TrimK9/K27 in the present study. The CENP-A antibody was a generous gift from Manuel Valdivia (Cadiz University, Spain) [83]. Antibodies to H3DimK4, H3TrimK9/K27 and CENP-A were raised in rabbits. Primary and secondary antibody incubations were in 1× PBS supplemented with 1% BSA (Sigma). Secondary antibodies were purchased from Jackson ImmunoResearch. After immunofluorescence detection, 20-50 spreads were captured and their positions on the slide recorded. Slides were subsequently hybridized with an appropriate alpha-satellite probe to detect transfected and endogenous alpha-satellite sequences. FISH was carried out using standard protocols.

### Replication timing assay

Cells were pulsed with 10 μM BrdU (Roche) in T25 cm^2 ^flasks for 2 h intervals. Following three PBS washes, medium supplemented with 50 μM thymidine (Sigma) was added. Cells were left in thymidine-containing medium until chromosome harvest. At appropriate intervals, colcemid was added to block cells in mitosis. Cells were harvested and fixed in 3:1 methanol/acetic acid. Metaphase spreads on microscope slides were baked for 1 h at 60°C. Primary anti-BrdU antibody (Roche) was added for 1 h at room temperature. Rhodamine-donkey-anti-mouse secondary antibodies (Jackson ImmunoResearch) were used to visualize sites of BrdU incorporation. Typically, 25 metaphase spreads were captured following BrdU detection and their coordinates on the slide recorded. Subsequent analyses with a D17Z1 probe were used to confirm identity of artificial or endogenous chromosomes.

## Figures and Tables

**Table 1:** Artificial chromosome segregation errors

		Number (%) of chromosome mis-segregation events*						
		
Line	Number of cells analyzed	Artificial	NDJ	Lag	Host 17	Host X	NDJ	Lag
17-B12	86	5/208 (2.4)	3	2	3/295 (1.0)		2	1
17-C20	224	91/745 (12.2)^†^	31	60	13/884 (1.5)		9	4
X-4^‡^	400	16/866 (1.8)	10	6		25/1,596 (1.6)	20	5
X-5^‡^	400	43/1,954 (2.2)	30	13		4/1,588 (0.2)	4	0

*Chromosome segregation errors (either artificial chromosomes or host chromosomes 17 or X) were nondisjunction (NDJ) or anaphase lag (Lag) events. ^†^The predominant artificial chromosome segregation error in 17-C20 was due to anaphase lag (66%, *n *= 91). ^‡^Data for X-4 and X-5 have been published [14]. Segregation errors that could not be classified (for example, 1:0) were excluded from these analyses.

**Table 2:** Chromatin formation on artificial chromosomes

Line	Artificial chromosomes		Heterochromatin		Euchromatin	
			
	Alpha-satellite	Size estimate*	H3 TrimK9/K27	HP1α	H3DimK4	^†^CENP-A
17-E29	D17Z1	1-3 Mb	-	(-)	+	+
						
17-D34	D17Z1	1-3 Mb	-	(-)	+	+
						
17-B12	D17Z1	3-10 Mb	+	+	+	+
17-C20^‡^	D17Z1	3-10 Mb	+	+	+	+
						
X-4	DXZ1	10-20 Mb	+	ND	+	ND
X-5	DXZ1	10-20 Mb	+	ND	+	ND
						
Host controls						
17 cen			+	+	-	+
X cen			+	ND	-	+

Summary of results obtained by immunofluorescence staining on metaphase chromosomes containing artificial chromosomes using antibodies to either heterochromatin (H3TrimK9/K27; HP1α) or euchromatin (H3DimK4) components (Figures 1-3). + positive staining; - signal not detectable; (-) weak staining comparable to general arm staining; ND, not done. *Comparison of alpha satellite signal intensities (using FISH analyses) on the artificial chromosomes with those of the relevant host centromere regions was used to estimate artificial chromosome sizes. ^†^CENP-A stains uniformly on artificial chromosomes and at a level comparable to the host staining level [12]. Controls, staining pattern at either host 17 or X centromere regions overlapping with D17Z1 or DXZ1 probes (respectively). ^‡^17-C20 contains mitotically unstable artificial chromosomes (Table 1).

**Table 3:** Replication timing of artificial chromosomes

	Artificial chromosomes		Replication timing							
			
			Early: 0-2 h S		Mid: 2-4 h S		Mid: 4-6 h S		Late: 6-8 h S	
			
Line	Chromatin composition*	Replication timing^†^	L	U	L	U	L	U	L	U
17-E29	Euchromatin	Early/mid	15	10	4	15	1	29	0	26
17-D34	Euchromatin	Early/mid	16	11	26	13	0	31	0	32
17-B12	Euchromatin/heterochromatin	Mid	2	24	14	15	9	19	0	30
17-C20	Euchromatin/heterochromatin	Mid	0	56	20	57	0	57	0	57
										
Controls										
17 cen	Heterochromatin	Mid	5	182	27	166	46	209	7	236
Yq	Heterochromatin	Mid/late	0	138	1	166	70	116	118	81

The number of either labeled (L) or unlabeled (U) artificial chromosomes in lines 17-E29, 17-D34, 17-B12 or 17-C20 or host control 17 centromere regions (17 cen) or Y long arm sequences (Yq) following BrdU detection at 2 h intervals in S phase is indicated in columns early, mid or late S phase. *Chromatin composition of artificial chromosomes in the four lines indicated or control host 17 centromere or Yq regions (see Table 2). Euchromatin: euchromatin present; heterochromatin depleted. Euchromatin/heterochromatin: both euchromatin and heterochromatin present. Heterochromatin: predominantly heterochromatin; euchromatin depleted. ^†^Predominant phase in S phase during which replication occurs: early/mid: first half (0-4 h) of S phase; mid S phase (2-6 h into S phase); mid to late S (4-8 h into S phase). Pooled data from all experiments were used to generate the numbers for the controls.
